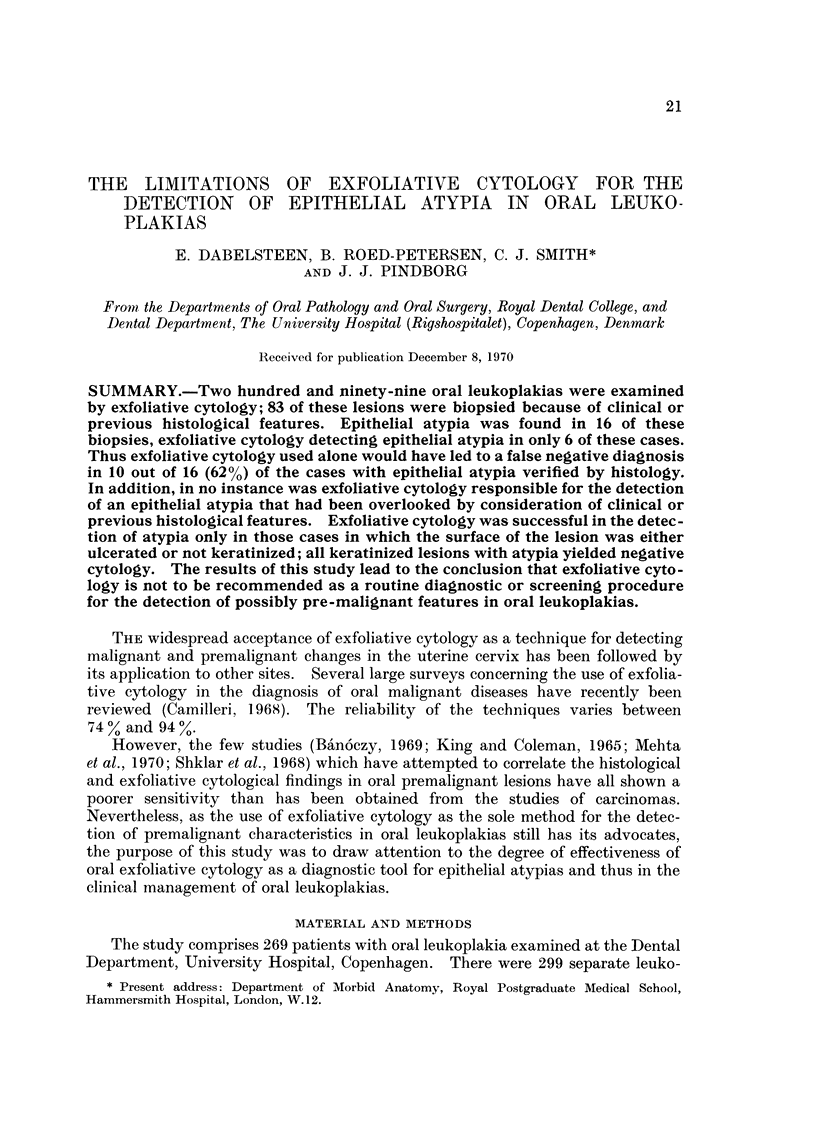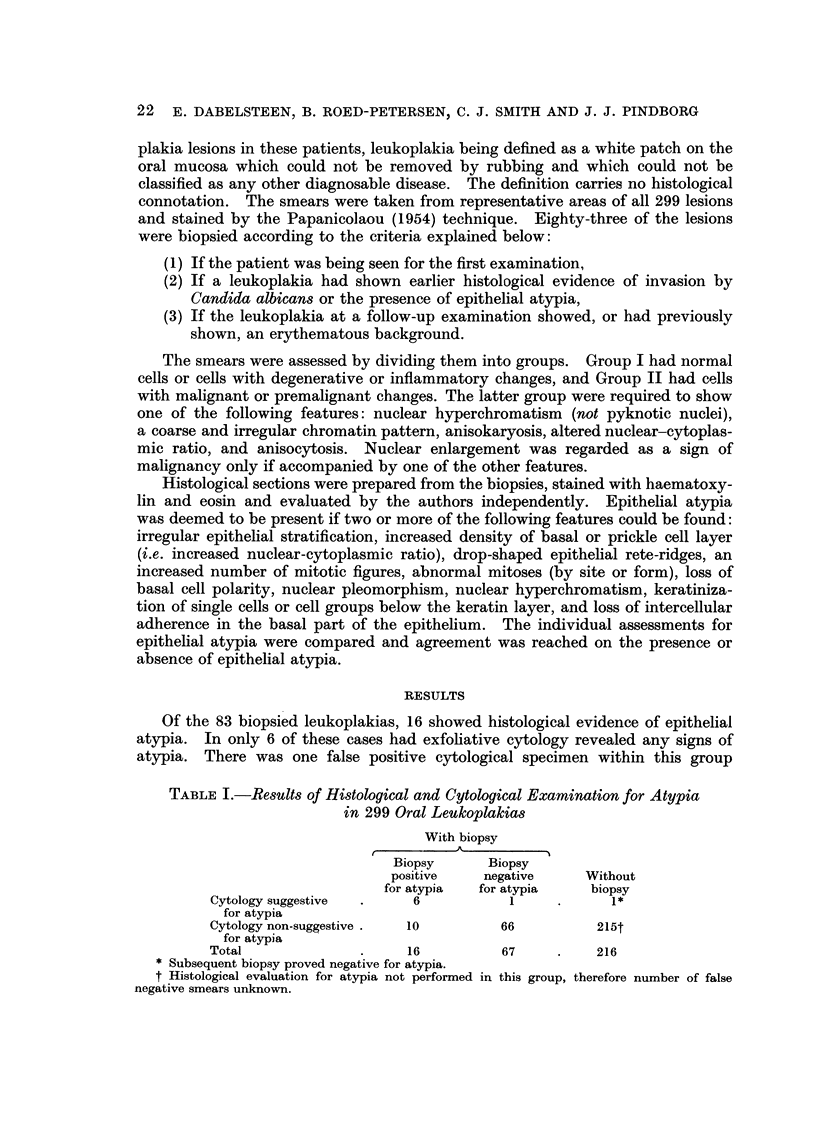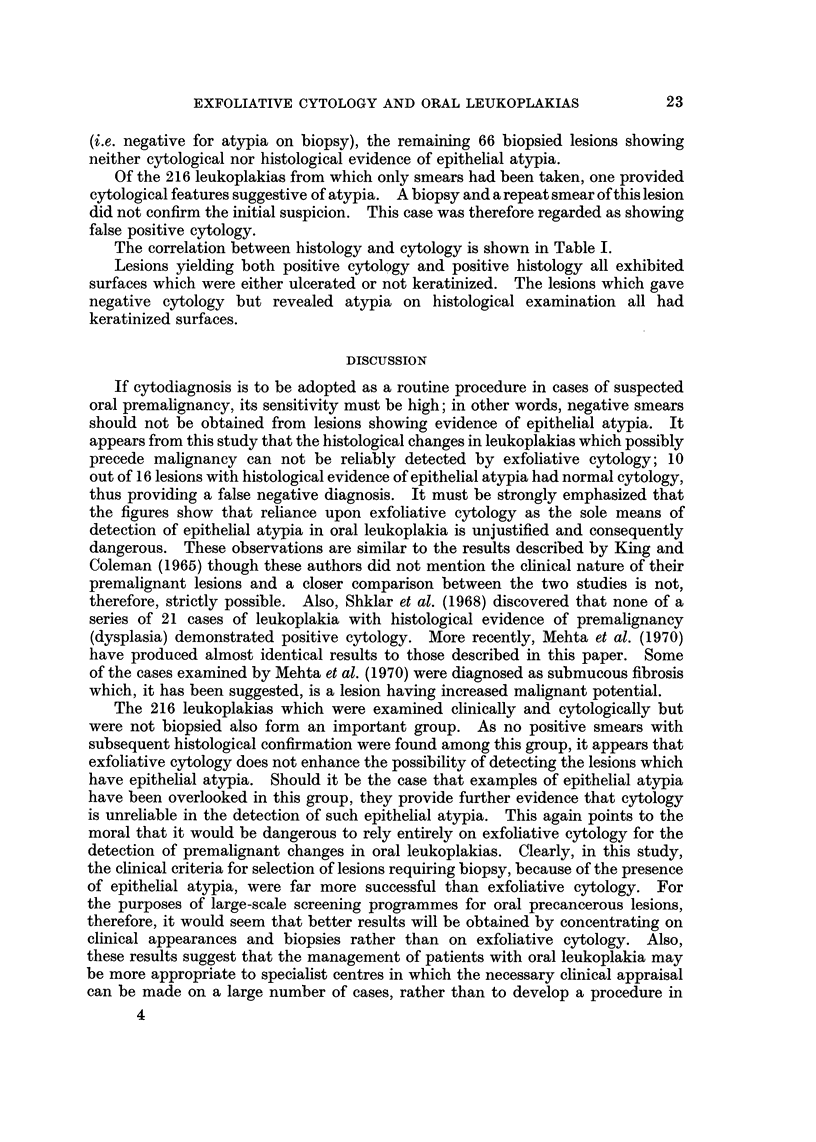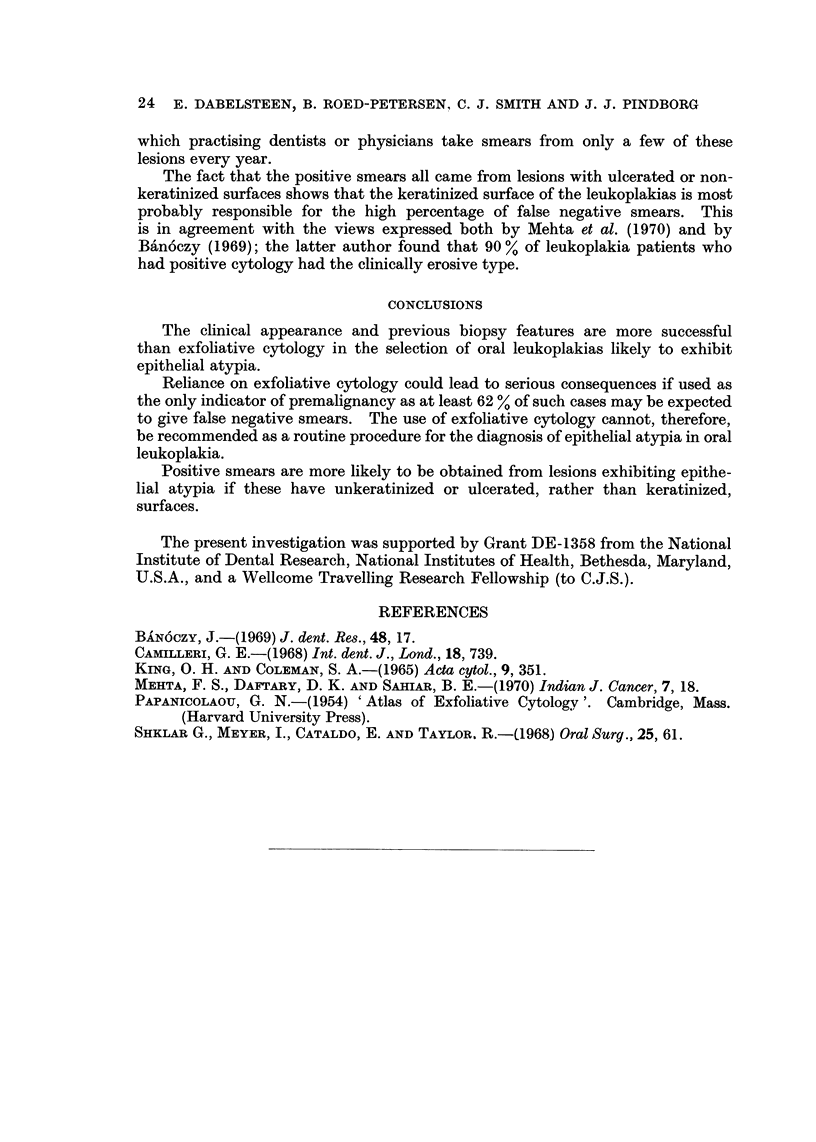# The Limitations of Exfoliative Cytology for the Detection of Epithelial Atypia in Oral Leukoplakias

**DOI:** 10.1038/bjc.1971.3

**Published:** 1971-03

**Authors:** E. Dabelsteen, B. Roed-Petersen, C. J. Smith, J. J. Pindborg

## Abstract

Two hundred and ninety-nine oral leukoplakias were examined by exfoliative cytology; 83 of these lesions were biopsied because of clinical or previous histological features. Epithelial atypia was found in 16 of these biopsies, exfoliative cytology detecting epithelial atypia in only 6 of these cases. Thus exfoliative cytology used alone would have led to a false negative diagnosis in 10 out of 16 (62%) of the cases with epithelial atypia verified by histology. In addition, in no instance was exfoliative cytology responsible for the detection of an epithelial atypia that had been overlooked by consideration of clinical or previous histological features. Exfoliative cytology was successful in the detection of atypia only in those cases in which the surface of the lesion was either ulcerated or not keratinized; all keratinized lesions with atypia yielded negative cytology. The results of this study lead to the conclusion that exfoliative cytology is not to be recommended as a routine diagnostic or screening procedure for the detection of possibly pre-malignant features in oral leukoplakias.


### THE LIMITATIONS OF EXFOLIATIVE CYTOLOGY FOR THE DETECTION OF EPITHELIAL ATYPIA IN ORAL LEUKOPLAKIAS

E. DABELSTEEN, B. ROED-PETERSEN, C. J. SMITH* AND J. J. PINDBORG

*From the Departments of Oral Pathology and Oral Surgery, Royal Dental College, and Dental Department, The University Hospital (Rigshospitalet), Copenhagen, Denmark*

Received for publication December 8, 1970

**SUMMARY.—Two hundred and ninety-nine oral leukoplakias were examined by exfoliative cytology; 83 of these lesions were biopsied because of clinical or previous histological features. Epithelial atypia was found in 16 of these biopsies, exfoliative cytology detecting epithelial atypia in only 6 of these cases. Thus exfoliative cytology used alone would have led to a false negative diagnosis in 10 out of 16 (62%) of the cases with epithelial atypia verified by histology. In addition, in no instance was exfoliative cytology responsible for the detection of an epithelial atypia that had been overlooked by consideration of clinical or previous histological features. Exfoliative cytology was successful in the detection of atypia only in those cases in which the surface of the lesion was either ulcerated or not keratinized; all keratinized lesions with atypia yielded negative cytology. The results of this study lead to the conclusion that exfoliative cytology is not to be recommended as a routine diagnostic or screening procedure for the detection of possibly pre-malignant features in oral leukoplakias.**

THE widespread acceptance of exfoliative cytology as a technique for detecting malignant and premalignant changes in the uterine cervix has been followed by its application to other sites. Several large surveys concerning the use of exfoliative cytology in the diagnosis of oral malignant diseases have recently been reviewed (Camilleri, 1968). The reliability of the techniques varies between 74% and 94%.

However, the few studies (Bánóczy, 1969; King and Coleman, 1965; Mehta *et al.*, 1970; Shklar *et al.*, 1968) which have attempted to correlate the histological and exfoliative cytological findings in oral premalignant lesions have all shown a poorer sensitivity than has been obtained from the studies of carcinomas. Nevertheless, as the use of exfoliative cytology as the sole method for the detection of premalignant characteristics in oral leukoplakias still has its advocates, the purpose of this study was to draw attention to the degree of effectiveness of oral exfoliative cytology as a diagnostic tool for epithelial atypias and thus in the clinical management of oral leukoplakias.

### MATERIAL AND METHODS

The study comprises 269 patients with oral leukoplakia examined at the Dental Department, University Hospital, Copenhagen. There were 299 separate leuko-

* Present address: Department of Morbid Anatomy, Royal Postgraduate Medical School, Hammersmith Hospital, London, W.12.

plakia lesions in these patients, leukoplakia being defined as a white patch on the oral mucosa which could not be removed by rubbing and which could not be classified as any other diagnosable disease. The definition carries no histological connotation. The smears were taken from representative areas of all 299 lesions and stained by the Papanicolaou (1954) technique. Eighty-three of the lesions were biopsied according to the criteria explained below:

(1) If the patient was being seen for the first examination,
(2) If a leukoplakia had shown earlier histological evidence of invasion by *Candida albicans* or the presence of epithelial atypia,
(3) If the leukoplakia at a follow-up examination showed, or had previously shown, an erythematous background.

The smears were assessed by dividing them into groups. Group I had normal cells or cells with degenerative or inflammatory changes, and Group II had cells with malignant or premalignant changes. The latter group were required to show one of the following features: nuclear hyperchromatism (*not* pyknotic nuclei), a coarse and irregular chromatin pattern, anisokaryosis, altered nuclear–cytoplasmic ratio, and anisocytosis. Nuclear enlargement was regarded as a sign of malignancy only if accompanied by one of the other features.

Histological sections were prepared from the biopsies, stained with haematoxylin and eosin and evaluated by the authors independently. Epithelial atypia was deemed to be present if two or more of the following features could be found: irregular epithelial stratification, increased density of basal or prickle cell layer (*i.e.* increased nuclear-cytoplasmic ratio), drop-shaped epithelial rete-ridges, an increased number of mitotic figures, abnormal mitoses (by site or form), loss of basal cell polarity, nuclear pleomorphism, nuclear hyperchromatism, keratinization of single cells or cell groups below the keratin layer, and loss of intercellular adherence in the basal part of the epithelium. The individual assessments for epithelial atypia were compared and agreement was reached on the presence or absence of epithelial atypia.

### RESULTS

Of the 83 biopsied leukoplakias, 16 showed histological evidence of epithelial atypia. In only 6 of these cases had exfoliative cytology revealed any signs of atypia. There was one false positive cytological specimen within this group

TABLE I.—*Results of Histological and Cytological Examination for Atypia in 299 Oral Leukoplakias*

| | With biopsy | | |
|---|---|---|---|
| | Biopsy positive for atypia | Biopsy negative for atypia | Without biopsy |
| Cytology suggestive for atypia | 6 | 1 | 1* |
| Cytology non-suggestive for atypia | 10 | 66 | 215† |
| Total | 16 | 67 | 216 |

* Subsequent biopsy proved negative for atypia.

† Histological evaluation for atypia not performed in this group, therefore number of false negative smears unknown.

(*i.e.* negative for atypia on biopsy), the remaining 66 biopsied lesions showing neither cytological nor histological evidence of epithelial atypia.

Of the 216 leukoplakias from which only smears had been taken, one provided cytological features suggestive of atypia. A biopsy and a repeat smear of this lesion did not confirm the initial suspicion. This case was therefore regarded as showing false positive cytology.

The correlation between histology and cytology is shown in Table I.

Lesions yielding both positive cytology and positive histology all exhibited surfaces which were either ulcerated or not keratinized. The lesions which gave negative cytology but revealed atypia on histological examination all had keratinized surfaces.

### DISCUSSION

If cytodiagnosis is to be adopted as a routine procedure in cases of suspected oral premalignancy, its sensitivity must be high; in other words, negative smears should not be obtained from lesions showing evidence of epithelial atypia. It appears from this study that the histological changes in leukoplakias which possibly precede malignancy can not be reliably detected by exfoliative cytology; 10 out of 16 lesions with histological evidence of epithelial atypia had normal cytology, thus providing a false negative diagnosis. It must be strongly emphasized that the figures show that reliance upon exfoliative cytology as the sole means of detection of epithelial atypia in oral leukoplakia is unjustified and consequently dangerous. These observations are similar to the results described by King and Coleman (1965) though these authors did not mention the clinical nature of their premalignant lesions and a closer comparison between the two studies is not, therefore, strictly possible. Also, Shklar *et al.* (1968) discovered that none of a series of 21 cases of leukoplakia with histological evidence of premalignancy (dysplasia) demonstrated positive cytology. More recently, Mehta *et al.* (1970) have produced almost identical results to those described in this paper. Some of the cases examined by Mehta *et al.* (1970) were diagnosed as submucous fibrosis which, it has been suggested, is a lesion having increased malignant potential.

The 216 leukoplakias which were examined clinically and cytologically but were not biopsied also form an important group. As no positive smears with subsequent histological confirmation were found among this group, it appears that exfoliative cytology does not enhance the possibility of detecting the lesions which have epithelial atypia. Should it be the case that examples of epithelial atypia have been overlooked in this group, they provide further evidence that cytology is unreliable in the detection of such epithelial atypia. This again points to the moral that it would be dangerous to rely entirely on exfoliative cytology for the detection of premalignant changes in oral leukoplakias. Clearly, in this study, the clinical criteria for selection of lesions requiring biopsy, because of the presence of epithelial atypia, were far more successful than exfoliative cytology. For the purposes of large-scale screening programmes for oral precancerous lesions, therefore, it would seem that better results will be obtained by concentrating on clinical appearances and biopsies rather than on exfoliative cytology. Also, these results suggest that the management of patients with oral leukoplakia may be more appropriate to specialist centres in which the necessary clinical appraisal can be made on a large number of cases, rather than to develop a procedure in which practising dentists or physicians take smears from only a few of these lesions every year.

The fact that the positive smears all came from lesions with ulcerated or non-keratinized surfaces shows that the keratinized surface of the leukoplakias is most probably responsible for the high percentage of false negative smears. This is in agreement with the views expressed both by Mehta *et al.* (1970) and by Bánóczy (1969); the latter author found that 90 % of leukoplakia patients who had positive cytology had the clinically erosive type.

### CONCLUSIONS

The clinical appearance and previous biopsy features are more successful than exfoliative cytology in the selection of oral leukoplakias likely to exhibit epithelial atypia.

Reliance on exfoliative cytology could lead to serious consequences if used as the only indicator of premalignancy as at least 62 % of such cases may be expected to give false negative smears. The use of exfoliative cytology cannot, therefore, be recommended as a routine procedure for the diagnosis of epithelial atypia in oral leukoplakia.

Positive smears are more likely to be obtained from lesions exhibiting epithelial atypia if these have unkeratinized or ulcerated, rather than keratinized, surfaces.

The present investigation was supported by Grant DE-1358 from the National Institute of Dental Research, National Institutes of Health, Bethesda, Maryland, U.S.A., and a Wellcome Travelling Research Fellowship (to C.J.S.).

### REFERENCES

Bánóczy, J.—(1969) *J. dent. Res.*, **48**, 17.

Camilleri, G. E.—(1968) *Int. dent. J., Lond.*, **18**, 739.

King, O. H. and Coleman, S. A.—(1965) *Acta cytol.*, **9**, 351.

Mehta, F. S., Daftary, D. K. and Sahiar, B. E.—(1970) *Indian J. Cancer*, **7**, 18.

Papanicolaou, G. N.—(1954) 'Atlas of Exfoliative Cytology'. Cambridge, Mass. (Harvard University Press).

Shklar G., Meyer, I., Cataldo, E. and Taylor, R.—(1968) *Oral Surg.*, **25**, 61.